# Health Seeking Behaviour Related to Hypertension Among Adults in Africa: A Scoping Review

**DOI:** 10.1002/puh2.70459

**Published:** 2026-11-13

**Authors:** Mustapha Sanyang, Hafiz T. A. Khan, Salim Vohra

**Affiliations:** ^1^ University of West London London UK; ^2^ Ministry of Health Banjul Gambia; ^3^ Professorial Fellow, Oxforf Institute of Population Aging University of Oxford London UK

**Keywords:** adults, Africa, health seeking behaviour, hypertension, management, prevention

## Abstract

**Background:**

Hypertension is a major public health challenge in Africa, yet evidence on health‐seeking behaviour and its determinants remains fragmented. This scoping review aimed to map the available evidence on health‐seeking behaviour and its determinants related to hypertension among adults in Africa.

**Methods:**

The scoping review was conducted in accordance with the Joanna Briggs Institute (JBI) methodological guidance for scoping reviews and reported in line with the Preferred Reporting Items for Systematic reviews and Meta‐Analyses extension for Scoping Reviews (PRISMA‐ScR). Seven electronic databases were searched for peer‐reviewed studies published between January 2010 and January 2025. Studies involving adults aged 18 years and older in African countries and addressing health‐seeking behaviour or its determinants in relation to hypertension were eligible for inclusion.

**Results:**

Sixteen studies from nine African countries were included. Healthcare‐seeking behaviours were influenced by socio‐economic status, cultural beliefs, social support, health system factors and knowledge of hypertension. Medical pluralism and delayed healthcare utilization were commonly reported.

**Conclusion:**

Hypertension‐related health‐seeking behaviour in Africa is shaped by individual, social, cultural and health‐system factors. Interventions that address these factors are needed to improve hypertension prevention, early detection, treatment uptake and control.

## Graphical abstract

This scoping review synthesized evidence on hypertension‐related health‐seeking behaviour among adults aged ≥18 years across Africa. Sixteen studies from nine African countries revealed that healthcare decisions are shaped by knowledge and awareness, socio‐economic status, social support, cultural beliefs and health‐system factors. The findings highlight widespread use of both formal and informal healthcare sources, including hospitals, pharmacies, traditional healers, herbal remedies and faith‐based care. Strengthening community engagement, health education, screening opportunities and access to affordable, quality healthcare may improve hypertension detection, treatment uptake, adherence and long‐term blood pressure control.

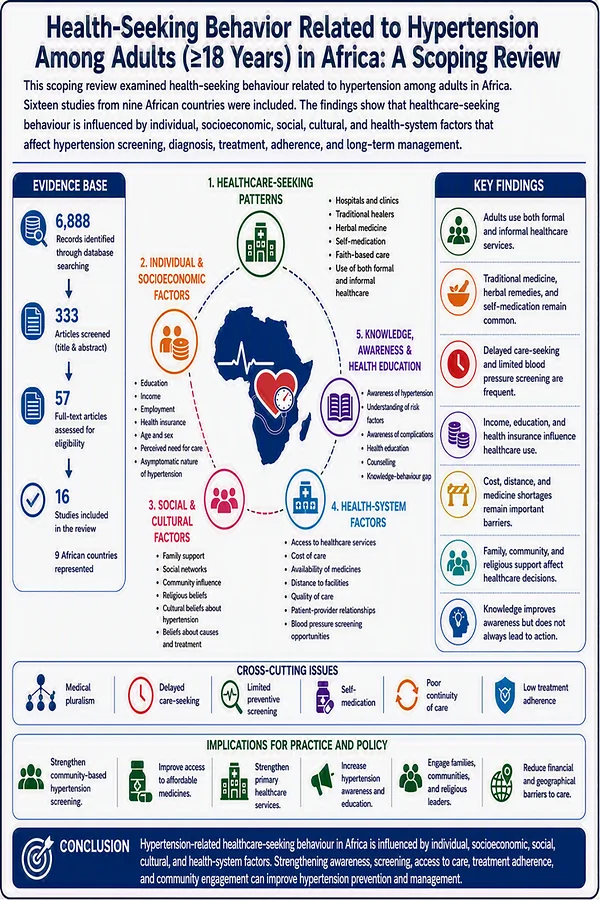

## 1 Introduction

Hypertension is one of the leading causes of cardiovascular disease worldwide, including stroke, coronary heart disease, heart failure and kidney disease [28]. Despite global and regional efforts, hypertension care remains inadequate. Globally, only 54% of adults with hypertension are diagnosed, 42% receive treatment, and just 21% achieve adequate blood pressure control [46]. Many individuals remain undiagnosed due to the often silent nature of the condition. Although hypertension is largely preventable and manageable through early detection, appropriate treatment and sustained control, but its prevalence continues to increase globally, with the greatest burden increasingly observed in low‐ and middle‐income countries [13, 39] estimated that 26.4% of the world's adult population had hypertension in 2000 and projected that this proportion would increase to 29.2%, representing approximately 1.56 billion adults, by 2025. This growing global burden is particularly concerning in Africa, where the epidemiological transition and changing population lifestyles have contributed to a substantial increase in hypertension. In 2013, approximately 74.7 million adults in Africa were estimated to have hypertension, with the number projected to increase to 125.5 million by 2025 [33]. More recent projections suggested that this burden could reach approximately 216.8 million by 2030 [4, 33]. The increasing burden is multifactorial, reflecting genetic, environmental, behavioural and socio‐economic factors that influence both the development of the condition and its prevention and management [47]. The growing burden of hypertension in Africa is occurring alongside rapid urbanization, population ageing, unhealthy dietary practices, physical inactivity and other lifestyle changes that have contributed to the increasing prevalence of non‐communicable diseases across the continent [38]. These challenges are compounded by weaknesses within health systems, including limited healthcare resources, inadequate screening services and low levels of awareness of hypertension, which can hinder prevention, early detection and effective management [12]. Consequently, hypertension remains a major contributor to morbidity and mortality in many African settings.

**What is already known**

- Hypertension is a major and growing public health problem in Africa, contributing substantially to cardiovascular disease, stroke and other related complications.
- Awareness, treatment and blood pressure control remain suboptimal, despite hypertension being largely preventable and treatable.
- Adults with hypertension commonly use both formal and informal healthcare sources to manage their condition.
- Delayed healthcare‐seeking, self‐medication and treatment interruption are common across African settings.

**What is new from this scoping review**

- This review synthesized evidence from 16 studies across nine African countries, providing one of the few continent‐focused reviews on hypertension‐related healthcare‐seeking behaviour.
- The review shows that healthcare‐seeking behaviour is influenced by the interaction of, individual factors, socio‐economic factors, cultural and religious beliefs, family and community influences and health‐system characteristics.
- Medical pluralism is common, with people using both conventional and non‐conventional medicines to manage their health.
- The asymptomatic nature of hypertension significantly contributes to delayed diagnosis and delayed treatment seeking.
- Knowledge does not consistently translate into healthcare‐seeking behaviour, highlighting a persistent knowledge–behaviour gap.
- Community and faith‐based structures can improve hypertension awareness, screening uptake, linkage to care and treatment adherence.
- Financial barriers such as medication costs, transportation expenses and consultation fees reduce access to care.
- Evidence on preventive healthcare‐seeking behaviour and routine hypertension screening among undiagnosed adults remains limited.

**Clinical implications**

- Clinicians should routinely assess patients for the use of non‐conventional medicines.
- Patient counselling should emphasize the asymptomatic nature of hypertension, importance of regular blood pressure monitoring, lifelong treatment adherence and prevention of complications.
- Family members should be involved in hypertension management plans whenever appropriate.
- Opportunistic blood pressure screening should be integrated into every healthcare encounter.

**Public health implications**

- Strengthen community‐based hypertension screening programmes.
- Improve access to affordable antihypertensive medications.
- Reduce transportation and financial barriers to care.
- Strengthen primary healthcare systems for long‐term hypertension management.
- Integrate religious leaders, community leaders and community health workers into hypertension awareness programmes.
- Develop culturally sensitive interventions that address misconceptions while promoting evidence‐based care.

The burden is further complicated by its often asymptomatic nature, with many individuals remaining unaware of their condition until serious complications develop [39]. This characteristic makes early detection and timely treatment particularly important for preventing adverse health outcomes. However, effective prevention and management extend beyond the availability of healthcare services. They also depend on individuals’ ability to recognize health problems, make decisions about when and where to seek care, access appropriate services and follow recommended treatment and lifestyle advice. Health‐seeking behaviour therefore represents an important component of the hypertension care pathway, influencing prevention, early detection, treatment and long‐term disease control.

In broader terms, health‐seeking behaviour refers to the actions individuals undertake to maintain or improve their health, prevent illness, recognize symptoms, seek appropriate healthcare, obtain treatment and follow health‐related advice [14]. Within this broader concept, healthcare‐seeking behaviour specifically refers to the process of seeking assistance from healthcare providers or healthcare systems for the diagnosis, treatment or management of a health condition [32]. In the context of hypertension, these behaviours may include participating in blood pressure screening, seeking care from healthcare providers when hypertension is suspected or diagnosed, initiating and continuing prescribed medication, adopting recommended lifestyle practices and attending follow‐up appointments [29].

Understanding these behaviours is particularly important in Africa, where formal healthcare systems often operate alongside traditional and faith‐based forms of care. These multiple sources of care may influence how individuals interpret hypertension and make decisions about where to seek care and access or continue treatment.

Existing evidence suggests that health‐seeking behaviour related to hypertension is influenced by several factors. Knowledge and awareness of hypertension have been associated with improved healthcare utilization and self‐management practices [1, 5, 29]. Cultural beliefs and perceptions about the causes and treatment of hypertension also shape healthcare decisions, including the use of traditional, faith‐based and formal healthcare services [15, 31, 35]. In addition, socio‐economic factors such as income, education and employment status, together with health system challenges, influence access to and utilization of hypertension‐related healthcare services [6, 20, 22, 23].

Although a growing number of studies have explored hypertension awareness, treatment, adherence and healthcare utilization in African countries, the available evidence remains scattered across different populations, settings and study designs. Most of the existing research has focused on individuals already diagnosed with hypertension or on specific aspects of healthcare utilization. Consequently, the broader patterns of health‐seeking behaviour related to hypertension among adults in African communities remain insufficiently understood. To our knowledge, no scoping review has comprehensively mapped and synthesized the available evidence on this topic across Africa.

Given the silent nature of hypertension and the fact that people seek care in different ways, it is important to understand how adults recognize hypertension‐related health needs, seek care, access services and engage in treatment and prevention practices. Mapping the available evidence can help identify the factors shaping these behaviours, highlight gaps in the existing literature, guide future research and inform strategies aimed at improving early detection, treatment uptake and hypertension control.

Therefore, this scoping review aimed to map the existing evidence on health‐seeking behaviour related to hypertension among adults aged 18 years and older in Africa and to identify the factors influencing the care‐seeking behaviour across the African continent.

## 2 Methods

This scoping review was conducted in accordance with the Joanna Briggs Institute (JBI) methodology for scoping reviews, [7]. The JBI methodology was used to guide the development of review questions, eligibility criteria, search strategy, study selection, data extraction and synthesis and presentation of the findings. The review was reported in accordance with the Preferred Reporting Items for Systematic reviews and Meta‐Analyses extension for Scoping Reviews (PRISMA‐ScR) guidelines to promote transparent and comprehensive reporting [43].

### 2.1 Review Questions

Following the JBI methodology, the review addressed the following questions:

1. What does the existing literature report about health‐seeking behaviour among adults aged 18 years and older in relation to hypertension across the African continent?
2. What are the key determinants influencing health‐seeking practices among adults in Africa in relation to hypertension?

### 2.2 Eligibility Criteria

The Population, Concept and Context (PCC) framework recommended by the JBI was used to define the eligibility criteria.

### 2.3 Population

The review included studies involving adults aged 18 years and older in Africa, including individuals with and without hypertension. This population was considered to capture health‐seeking behaviours related to hypertension prevention and management.

### 2.4 Concept

The review included studies examining health‐seeking behaviour, healthcare‐seeking practices and determinants influencing these behaviours in relation to hypertension prevention and management. Qualitative, quantitative and mixed‐methods studies were eligible for inclusion.

### 2.5 Context

Studies conducted in African countries were included to capture evidence from diverse healthcare systems and sociocultural contexts across the continent. Only peer‐reviewed studies published in English between January 2010 and January 2025 were included.

### 2.6 Search Strategy

A comprehensive literature search was conducted across seven electronic databases, PubMed, ScienceDirect, CINAHL, Embase, Scopus, Academic Search Elite and PsycINFO.

The search was limited to peer‐reviewed articles published between January 2010 and January 2025 to capture evidence reflecting contemporary health‐seeking behaviours and healthcare contexts. A University of West London librarian assisted in developing the search strategies. Key concepts derived from the review questions guided the development of the search strategy. For PubMed, Medical Subject Headings (MeSH) terms were combined with free‐text keywords to ensure a comprehensive and sensitive search. For the other databases, free‐text keywords were used according to their respective indexing systems and search functionalities. The search strategies were subsequently adapted to accommodate the specific indexing requirements and search functionalities of each database.

Searches were conducted across the seven databases between 24 and 29 March 2025. The complete database‐specific search strategies are presented in Table 1. A total of 6888 records were identified across all databases: PubMed (*n* = 3864), ScienceDirect (*n* = 2190), CINAHL (*n* = 715), Embase (*n* = 50), Scopus (*n* = 40), Academic Search Elite (*n* = 23) and PsycINFO (*n* = 6).

**TABLE 1:** Keywords for database and article identifications.

Database	Search terms and keywords	Number of articles	Date accessed
Embase	(‘Health‐seeking practice’ OR ‘health‐seeking behaviour’ OR ‘health seeking’ OR ‘healthcare seeking behaviour’ OR ‘healthcare utilization’ OR ‘health seeking behaviour’)) AND (adults OR adult OR ‘middle aged’ OR elderly OR ‘older person’ OR ‘young adult’) AND (hypertension OR ‘high blood pressure’ OR ‘elevated blood pressure’ OR HTN OR hypertensive) AND (Africa OR ‘Sub‐Saharan Africa’ OR Gambia OR Nigeria OR Senegal OR Mali OR Burkina Faso OR Kenya OR Zimbabwe OR Tanzania OR ‘South Africa’ OR Ghana OR Eritrea OR Egypt OR Sudan OR Ethiopia OR Rwanda OR Chad OR Libya OR Algeria OR Congo)	50	29/03/25
PubMed	(‘Health behaviour’ [Mesh] OR ‘health services accessibility’ [Mesh] OR ‘health‐seeking practice’ OR ‘health‐seeking behaviour’ OR ‘health seeking’ OR ‘healthcare seeking behaviour’ OR ‘healthcare utilization’)) AND (’Adult‘ [Mesh] OR adult * OR adults OR ‘elderly’) AND (‘hypertension’ [Mesh] OR hypertension OR hypertensive OR ‘high blood pressure’ OR ‘elevated blood pressure’) AND (‘Africa’ [Mesh] OR Africa OR ‘Sub‐Saharan Africa’ OR Gambia OR Nigeria OR Senegal OR Mali OR ‘Burkina Faso’ OR Kenya OR Zimbabwe OR Tanzania OR ‘South Africa’ OR Ghana OR Eritrea OR Egypt OR Sudan OR Ethiopia OR Rwanda OR Chad OR Libya OR Algeria OR Congo)	3864	26/03/25
PsycINFO	(‘Health‐seeking practice’ OR ‘health‐seeking behaviour’ OR ‘health seeking’ OR ‘healthcare seeking behaviour’ OR ‘healthcare utilization’ OR ‘health seeking behaviour’ OR ‘health literacy’)) AND (adults OR adult OR ‘middle aged’ OR elderly OR ‘older person’ OR ‘young adult’) AND (hypertension OR ‘high blood pressure’ OR ‘elevated blood pressure’ OR HTN OR hypertensive) AND (Africa OR ‘Sub‐Saharan Africa’ OR Gambia OR Nigeria OR Senegal OR Mali OR Burkina Faso OR Kenya OR Zimbabwe OR Tanzania OR ‘South Africa’ OR Ghana OR Eritrea OR Egypt OR Sudan OR Ethiopia OR Rwanda OR Chad OR Libya OR Algeria OR Congo)	6	24/03/25
CINAHL	(‘Health‐seeking practice’ OR ‘health‐seeking behaviour’ OR ‘health seeking’ OR ‘healthcare seeking behaviour’ OR ‘healthcare utilization’ OR ‘health seeking behaviour’)) AND (adults OR adult OR ‘middle aged’ OR elderly OR ‘older person’ OR ‘young adult’) AND (hypertension OR ‘high blood pressure’ OR ‘elevated blood pressure’ OR HTN OR hypertensive) AND (Africa OR ‘Sub‐Saharan Africa’ OR Gambia OR Nigeria OR Senegal OR Mali OR Burkina Faso OR Kenya OR Zimbabwe OR Tanzania OR ‘South Africa’ OR Ghana OR Eritrea OR Egypt OR Sudan OR Ethiopia OR Rwanda OR Chad OR Libya OR Algeria OR Congo)	715	24/03/25
Science Direct	(‘health‐seeking practice’ OR ‘health‐seeking behaviour’ OR ‘health seeking’ OR ‘healthcare seeking behaviour’ OR ‘healthcare utilization’ OR ‘health seeking behaviour’) AND (adults OR adult OR ‘middle aged’ OR elderly OR ‘older person’ OR ‘young adult’) AND (hypertension OR ‘high blood pressure’ OR ‘elevated blood pressure’ OR HTN OR hypertensive) AND (Africa OR ‘Sub‐Saharan Africa’ OR Gambia OR Nigeria OR Senegal OR Mali OR Burkina Faso OR Kenya OR Zimbabwe OR Tanzania OR ‘South Africa’ OR Ghana OR Eritrea OR Egypt OR Sudan OR Ethiopia OR Rwanda OR Chad OR Libya OR Algeria OR Congo)	2190	27/03/25
Scopus	(‘Health‐seeking practice’ OR ‘health‐seeking behaviour’ OR ‘health seeking’ OR ‘healthcare seeking behaviour’ OR ‘healthcare utilization’ OR ‘health seeking behaviour’) AND (adults OR adult OR ‘middle aged’ OR elderly OR ‘older person’ OR ‘young adult’) AND (hypertension OR ‘high blood pressure’ OR ‘elevated blood pressure’ OR HTN OR hypertensive) AND (Africa OR ‘Sub‐Saharan Africa’ OR Gambia OR Nigeria OR Senegal OR Mali OR Burkina Faso OR Kenya OR Zimbabwe OR Tanzania OR ‘South Africa’ OR Ghana OR Eritrea OR Egypt OR Sudan OR Ethiopia OR Rwanda OR Chad OR Libya OR Algeria OR Congo)	40	27/3/25
Academic Search Elite	(‘Health‐seeking practice’ OR ‘health‐seeking behaviour’ OR ‘health seeking’ OR ‘healthcare seeking behaviour’ OR ‘healthcare utilization’ OR ‘health seeking behaviour’) AND (adults OR adult OR ‘middle aged’ OR elderly OR ‘older person’ OR ‘young adult’) AND (hypertension OR ‘high blood pressure’ OR ‘elevated blood pressure’ OR HTN OR hypertensive) AND (Africa OR ‘Sub‐Saharan Africa’ OR Gambia OR Nigeria OR Senegal OR Mali OR Burkina Faso OR Kenya OR Zimbabwe OR Tanzania OR ‘South Africa’ OR Ghana OR Eritrea OR Egypt OR Sudan OR Ethiopia OR Rwanda OR Chad OR Libya OR Algeria OR Congo)	23	24/03/25

### 2.7 Exclusion Criteria

Studies were excluded if they focused exclusively on pregnant women, individuals younger than 18 years or populations outside Africa; were conference abstracts; or did not address health‐seeking behaviour or determinants related to health seeking behavior related hypertension. Editorials, commentaries, opinion pieces, protocols, theses, dissertations and review articles were also excluded because they did not provide primary empirical evidence directly relevant to the review objectives. Grey literature was excluded because the review was restricted to peer‐reviewed publications.

### 2.8 Study Selection

Following the JBI methodology, all identified records were screened against the predefined inclusion and exclusion criteria. The screening process consisted of three stages namely references screening, title and abstract screening, followed by full‐text assessment of potentially relevant articles. Studies that met all eligibility criteria were included in the review. The study selection process was documented using a PRISMA‐ScR flow diagram, which presents the number of records identified, screened, assessed for eligibility, excluded and included in the review. The characteristics of the selection process are presented in Figure 1.

**FIGURE 1:**
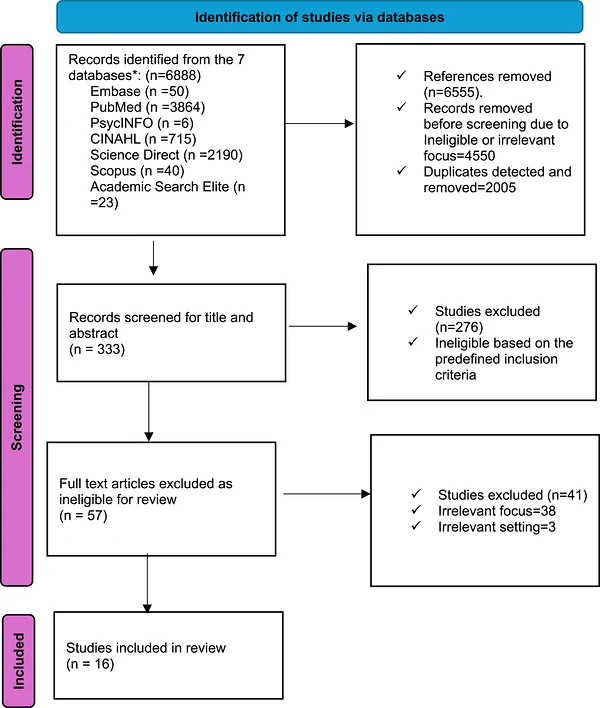
PRISMA flow diagram of scoping review process.

A total of 6888 records were identified through database searching. The first and second reviewers collaboratively imported all retrieved records into RefWorks and conducted an initial screening to remove duplicate citations and records that were clearly irrelevant to the review question. This process was guided by the predefined inclusion and exclusion criteria to ensure that only potentially relevant studies progressed to the next stage of screening. Following the removal of duplicates and obviously ineligible records, 333 peer‐reviewed articles remained and were considered eligible for title and abstract screening.

The two reviewers, who both had access to the shared University of West London OneDrive repository and RefWorks database, independently screened the titles and abstracts of the 333 articles against the eligibility criteria. Regular discussions were held throughout the screening process to ensure consistency in the application of the inclusion and exclusion criteria. During this stage, 276 articles were excluded because they did not address health‐seeking behaviour related to hypertension, majority focused on unrelated health conditions, involved ineligible populations or were otherwise inconsistent with the objectives of the review. Consequently, 57 articles were retained for full‐text assessment.

The full texts of these 57 articles were then independently reviewed by both reviewers using the predefined eligibility criteria. Reviewer 1 identified 19 potentially eligible studies, whereas Reviewer 2 identified 17 potentially eligible studies. Comparison of the reviewers’ decisions showed that 16 studies were independently identified by both reviewers and met all eligibility requirements for inclusion in the review. Any disagreements were discussed and resolved through consensus, ensuring transparency, consistency and methodological rigour throughout the selection process.

Among the studies identified exclusively by Reviewer 1, three were excluded because they were conducted outside Africa and therefore did not satisfy the geographical eligibility criterion. Similarly, one study identified exclusively by Reviewer 2 was excluded because it focused on cervical cancer rather than hypertension. Overall, 41 full‐text articles were excluded.

Consequently, 16 studies met all inclusion criteria and were included in the final scoping review. The systematic and collaborative screening process helped ensure that the final body of evidence was directly relevant to the review question and reflected the available literature on health‐seeking behaviour related to hypertension among adults in Africa. The complete study selection process is illustrated in the PRISMA‐ScR flow diagram (Figure 1).

### 2.9 Data Extraction

Data were independently extracted by two reviewers using a standardized data charting form. Extracted information included study characteristics, such as author(s), year of publication, study design, country or region, study setting and sample size, as well as key findings relevant to the review objectives. The characteristics of the included studies are presented in Table 2

**TABLE 2:** Articles included in the review process.

Study	Country	Study design	Sample size	Sampling technique	Study location	Study settings	Population type	Study objective	Findings	Recommendations
Tusubira et al. [44]	Uganda	Quantitative, cross‐sectional study	385	Systematic random sampling selection was used	Rural	Facility‐based study	Hypertensive patients	To describe and compare self‐care practices and healthcare‐seeking behaviours among patients with hypertension (HTN), diabetes mellitus (DM) and coexisting hypertension and diabetes (HTN/DM) in rural Uganda	Participants with diabetes (alone or combined with hypertension) had significantly more clinic visits than those with hypertension alone Medication adherence was significantly higher among participants with diabetes than those with hypertension alone. Exposure to self‐care education was substantially greater among diabetes patients compared to hypertension‐only patients Health‐related quality of life analyses showed that Women were more likely to experience challenges with self‐care Adults older than 65 years had significantly greater difficulties with self‐care activities	Strengthen self‐care education programmes for patients with hypertension, as they demonstrated lower exposure to self‐management education than diabetes patients Increase efforts to improve medication adherence and self‐monitoring practices among individuals with hypertension Develop targeted interventions for older adults and women who experience greater self‐care challenges Promote integrated NCD care models that combine management of hypertension and diabetes while enhancing self‐care support
Opare‐Addo et al. [34]	Ghana	Quantitative, cross‐sectional study	684	Multistage sampling strategy was used for this study	Rural	Community‐based study	Adults 18+	To determine the prevalence of hypertension and diabetes in rural districts in the Ashanti region of Ghana and to determine factors that influence utilization of healthcare services	The public hospitals are the preferred choice of health facilities for patients with hypertension and diabetes in the rural districtsThe main reasons for choosing a healthcare facility were as follows: Perceived quality of care Proximity/distance to the facility Predisposing factors significantly associated with healthcare facility preference included: Age Marital status Educational level Occupation Educational attainment significantly reduced the likelihood of seeking care from over‐the‐counter medicine shops (OTCMS) and health centres compared with hospitals. Income level, socio‐economic status and health insurance enrolment showed associations with facility preference, although they were not significant predictors in the final multinomial regression model	Strengthen public hospitals and health centres, as these are the preferred healthcare facilities for rural patients with hypertension and diabetes. Improve the quality of healthcare services, as perceived quality strongly influences healthcare utilization. Expand community‐based screening and early detection programmes for hypertension and diabetes to improve diagnosis and management. Develop targeted interventions to address the growing burden of non‐communicable diseases in rural Ghana Enhance health education initiatives aimed at improving healthcare‐seeking behaviour and encouraging timely utilization of formal healthcare services. Continue strengthening healthcare accessibility in rural communities through affordable, high‐quality and geographically accessible services
Nonterah et al. [30]	Ghana	Quantitative, cross‐sectional study	631	Voluntary selection of participants/convenience sampling according to the article	Rural	Facility‐based study (faith‐based centre	Community participants	To investigate the role of faith‐based centres (FBCs) in screening for hypertension and Type 2 diabetes mellitus (T2DM), linking suspected cases to healthcare facilities and assessing the hypertension and diabetes care cascade in the Kassena‐Nankana Districts of Northern Ghana	Faith‐based centres have the potential to enhance screening, linkage to the healthcare system and management of hypertension and Type 2 diabetes mellitus. This improvement over the routine system could lead to earlier diagnosis, a reduction in complications, decreased premature mortality from cardiovascular diseases	Integrate faith‐based centres into national hypertension and diabetes screening programmes to improve early detection and referral. Strengthen collaboration between faith leaders and healthcare providers to enhance health promotion and treatment adherence Improve public awareness, diagnosis, treatment and control of hypertension and diabetes through community‐based interventions
Musinquzi et al. [29]	Uganda	Qualitative study	40	Purposive sampling	Rural	Community‐based study	Community hypertensive patients	To explore factors influencing compliance and health seeking behaviour	Most participants used multiple healthcare pathways for hypertension management, including public health facilities, private clinics, pharmacies, self‐medication and traditional herbal remedies Hypertension control was poor Self‐medication and obtaining antihypertensive medications from drug shops, often without prescriptions, were common practices. Herbal medicine use was widespread, including remedies such as garlic, ginger, moringa, aloe vera and local herbal mixtures Regular blood pressure monitoring was uncommon because of limited access to blood pressure measurement services **Health system factors** Key barriers included: Frequent shortages of medicines and diagnostic equipment Inadequate healthcare personnel Long waiting times Poor staff attitudes and perceived low quality of care Limited availability of specialized hypertension services **Patient factors** Health‐seeking behaviour was influenced by Awareness of hypertension status Perceived severity of symptoms Beliefs about the effectiveness of treatment Concerns about medication side effects Fear of lifelong dependence on antihypertensive medication **Socio‐economic and structural factors** Poverty, transport costs, distance to health facilities and competing household financial priorities limited healthcare access Family support positively influenced treatment adherence and clinic attendance	Strategies to address the multifactorial dimension that affect health seeking behaviour are needed to improve hypertension control in this population Strengthening hypertension services through improved availability of medications, equipment and trained healthcare personnel. Increase community‐based hypertension screening and routine blood pressure monitoring services. Improve patient education on hypertension, including the importance of long‐term treatment adherence and blood pressure control. Address barriers related to transport, healthcare costs and access to services, particularly for rural populations
Lulebo et al. [27]	DRC Congo	Quantitative, cross‐sectional study	280	Convenience sampling	Urban	Facility‐based study	Adult 18+ hypertensive	To determine the prevalence and factors associated with the use of complementary and alternative medicine	The use of complementary alternative medicines for hypertensive patients is a major problem	Antihypertensives with fewer side effects must be emphasized. Religious leaders must become involved in the communication for behavioural change activities to improve the quality of life for hypertensive patients
Liwa et al. [26]	Tanzania	Mixed method	213	Convenience sampling	Urban	Facility‐based study	Hospitalized patients with hypertension	To determine prevalence, pattern and correlates of herbal and alternative medicine use in Tanzanian adults hospitalized with hypertension	The use of traditional medicines is very common among patients with hypertension. Adults from low socio‐economic status, those with misunderstandings about hypertension, and those without health insurance were more likely to take herbs	Healthcare providers should routinely inquire about herbal medicine use among patients with hypertension. Promote open, non‐judgmental communication between healthcare workers and patients regarding traditional medicine use. Provide patient education on hypertension, medication adherence, and potential herb–drug interactions. Improve access to health insurance and affordable healthcare services, particularly for low socio‐economic groups Future investigations should focus on identifying the magnitude of the problem in the community level, traditional healers’ knowledge about hypertension, and the timing of initiation of herbal use and alternative therapies
Konlan et al. [25]	Ghana	Quantitative, cross‐sectional study	294	Systematic sampling selection	Peri‐urban	Community‐based study	Adults	To assess the practices of adults in a peri‐urban community in the Ho Municipality of the Volta region on the prevention of hypertension	Hypertension prevalence was 18.7%; medication adherence among hypertensive participants was suboptimal despite high prescription rates Herbal medicine use was common, often alongside prescribed medication Participants generally reported positive lifestyle practices and high willingness to adopt healthier behaviours	Recommendations focused on health education, lifestyle modification, medication adherence and safe use of complementary therapies
Ketor et al. [24]	Ghana	Mixed method	295	Simple random sampling	Rural	Community‐based study	Adults	To determine the factors influencing health‐seeking behaviour among residents of the Jasikan District, Ghana, and to assess the importance of well‐being clinics in influencing healthcare‐seeking decisions	More than half of respondents used public healthcare facilities as their first point of contact when ill A large majority demonstrated **appropriate health‐seeking behaviour** Significant factors associated with health‐seeking behaviour included: **Age** **Health insurance status** **Religion** Important healthcare service characteristics influencing provider choice included: Affordable services (**59.9%**) Availability of medicines (**54.5%**) Good quality services (**52.0%**) Proximity of healthcare facilities Females were more likely to seek appropriate healthcare than males Severity of illness, perceived quality of care, professionalism and availability of trained staff influenced healthcare facility selection Most respondents supported the establishment of a well‐being clinic, citing benefits such as routine health checks, health status awareness, accessibility and early disease detection	Healthcare managers should prioritize: Affordable services Availability of medicines High‐quality healthcare delivery as these strongly influence healthcare utilization All health facilities should establish and operationalize **well‐being clinics** for routine screening and preventive care Health insurance coverage should be expanded to support well‐being clinic services and reduce financial barriers to healthcare access
Karinja et al. [22]	Kenya	Quantitative, cross‐sectional study	1100	Convenience sampling	Rural	Facility‐based study	Hypertensive adults	To examine healthcare‐seeking dynamics and factors associated with health‐seeking behaviour among adults diagnosed with diabetes mellitus and/or hypertension in selected rural communities in Kenya	Approximately more than half of the participants reported having regular scheduled clinic visits, indicating generally good health‐seeking behaviour Most participants were diagnosed and received treatment through **public health facilities** Positive predictors of appropriate health‐seeking behaviour included: Having social support during illness and treatment Good self‐rated health status Previous hospital admission within the last year. Negative predictors of appropriate health‐seeking behaviour included: Alcohol consumption Seeking treatment from pharmacies or chemists instead of healthcare facilities. Financial constraints, feeling better, long distances to health facilities and perceptions that the disease was not serious were common reasons for treatment discontinuation or non‐initiation	Strengthen public healthcare facilities to improve access and continuity of care for patients with diabetes and hypertension. Increase patient education and counselling on the chronic nature of hypertension and diabetes and the importance of long‐term treatment adherence. Expand health insurance coverage to reduce financial barriers to care. Enhance community‐based support systems and social support networks to improve treatment adherence and clinic attendance Strengthen and expand the role of community health volunteers (CHVs) in household education, follow‐up and chronic disease management
Jobe et al. [21]	Gambia	Quantitative, cross‐sectional study	9171	Multistage sampling strategy	Urban–rural	Community‐based study	Adults 35+	To evaluate the hypertension care cascade (prevalence, awareness, treatment and control) among adults aged 35 years and above in the Gambia and identify socio‐demographic factors associated with gaps in hypertension care	Hypertension prevalence was 47.0%, indicating a substantial burden among middle‐aged and older adults Among people with hypertension: 54.7% were aware of their diagnosis 32.5% were receiving treatment Only 10.0% had controlled blood pressure Women demonstrated better performance across the hypertension care cascade than men, with higher awareness, treatment and control rates Older age, female sex and higher BMI were associated with greater awareness of hypertension. Being employed was associated with greater likelihood of receiving treatment The largest loss in the care cascade occurred between having hypertension and being diagnosed, indicating a major challenge in early detection and screening No significant differences in hypertension care outcomes were observed between urban and rural populations	Implement a comprehensive national hypertension prevention and control strategy focused on improving all stages of the care cascade Increase public awareness campaigns on hypertension, healthy diets, physical activity and cardiovascular risk reduction. Strengthen community‐based hypertension screening programmes to improve early diagnosis Improve availability of blood pressure monitoring equipment and antihypertensive medications in healthcare facilities. Conduct further research to identify barriers to treatment adherence and blood pressure control among treated patients
Hudges et al. (2021)	South Africa	Quantitative, cross‐sectional study	721	Multistage sampling strategy	Urban–rural	Community‐based study	Adults 30+ years older	To identify the use of traditional medicines, we documented the prevalence of specific health conditions, the use of THM and the influences and reasons for using indigenous treatment practices	Non‐communicable diseases were more prevalent in urban communities with hypertension being the most prevalent. There is widespread use of traditional herbal medicine across South Africa. Rural residents and individuals of low social economic status rely more on traditional herbal medicine Rural residents had more than 12 times higher odds of using THM compared with urban residents after adjustment for socio‐demographic factors	They recommended for the need of systematic and larger studies to understand the magnitude and predictors of variation in access to and use of healthcare services, as these are critical to developing effective culturally appropriate interventions for approaching hard reach patients
Gebrezgi et al. [17]	Eritrea	Qualitative study	48	Convenience sampling	Urban	Facility‐based study	Hypertensive patients	To identify barriers and facilitators influencing hypertension management from the perspectives of hypertensive patients attending follow‐up clinics in Asmara, Eritrea	Barriers to hypertension management included financial difficulties, stress, misconceptions about physical activity, traditional medicine use, low community awareness, stigma and limited health promotion activities Facilitators included good patient knowledge, strong family support, positive healthcare provider relationships and free access to antihypertensive medication	Counselling patients about adherence to medication, strengthening family and government support and empowering families and the community with appropriate knowledge of hypertension management could potentially help in an individual adherence
Faronbi et al. [16]	Nigeria	Quantitative, cross‐sectional study	306	Multistage sampling strategy	Urban	University (urban)	University staff	The study assessed knowledge of hypertension risk factors, blood pressure (BP) monitoring practices and health‐seeking behaviours among university staff in Nigeria	Participants demonstrated moderate to good knowledge of hypertension risk factors, but regular BP monitoring practices were generally poor Many respondents relied on patent medicine stores and self‐management approaches when elevated blood pressure was detected Knowledge, age and socio‐economic status were associated with BP monitoring behaviour	Measures should be put in place to ensure that workers participate in regular blood pressure screening; this will help in prevention and early detection and better prognosis of hypertension
Bamgboye et al. [10]	Nigeria	Quantitative, cross‐sectional study	762	Multistage sampling strategy	Urban–rural	Community‐based study	Adults with hypertension	To assess the treatment seeking behaviour and associated factors among adults with high blood pressure from three ethnic groups in Nigeria	Adults with high blood pressure in this study practise orthodox treatment as first choice of treatment following diagnosis. Nearly half of the participants (48.3%) were unaware they had high blood pressure before the survey About half (**48.2%**) had not visited a healthcare facility within the previous year Blood pressure control was suboptimal, with only about **40.4%** of known hypertensive participants achieving controlled blood pressure. Significant predictors of orthodox treatment‐seeking behaviour included: Female gender Availability of medicines, healthcare personnel and diagnostic services Perceived good quality of healthcare services	People with high blood pressure need to be encouraged to continue to practise orthodox treatment throughout their life course of hypertension management
Azu et al. [9]	Nigeria	Quantitative, cross‐sectional study	2983	House to house interview	Abia State (community survey)	Cross‐sectional community survey	Adults 18+ years	To determine risk factors that affect care‐seeking behaviours and the association of those factors with undiagnosed hypertension in Southern Nigeria, predominantly comprised of the Igbo ethnic group	Their findings revealed that the age, sex, marital status, education, location and income were associated with care‐seeking behaviour	Strengthen routine hypertension screening in healthcare facilities and community settings Develop targeted interventions addressing socio‐demographic factors associated with poor healthcare‐seeking behaviour Improve blood pressure screening practices during routine healthcare visits to reduce undiagnosed hypertension and related complications Enhance early detection and prevention strategies, particularly among high‐risk groups such as younger males and individuals with obesity
Aboyade et al. [3]	South Africa	Quantitative, cross‐sectional study	250	Purposive sampling	Peri‐urban	Community‐based study	Women 50+ years older	To understand health‐seeking behaviours, healthcare practices and traditional herbal medicine (THM) use among older women living with non‐communicable diseases	Self‐reported non‐communicable diseases with comorbidities were prevalent among the participants in the study. Most of the participants utilized state‐owned clinics and hospitals for management of their chronic conditions. Traditional herbal medicine use was not very common	Improve communication between healthcare providers and patients regarding herbal medicine use Educate healthcare workers about medical pluralism and potential herb–drug interactions. Encourage patients to disclose traditional medicine use during consultations. Increase awareness of safe management of chronic diseases among older women with multiple conditions

### 2.10 Data Analysis and Presentation

The findings were synthesized using a narrative descriptive approach, focusing on health‐seeking behaviour related to hypertension. The findings were organized thematically to identify common patterns across the included studies, variations between settings and gaps in the existing evidence. A meta‐analysis was not undertaken because the purpose of the scoping review was to map the breadth, range and nature of the available evidence rather than to estimate pooled effects.

## 3 Results

### 3.1 Characteristics of Included Studies

The review included 16 studies conducted across nine African countries. Ghana contributed four studies, Nigeria three, South Africa two and Uganda two. Single studies were conducted in Tanzania, Kenya, The Gambia, the Democratic Republic of Congo and Eritrea.

Most studies employed quantitative designs (*n* = 12), whereas two were qualitative and two used mixed methods of designs. Sample sizes ranged from 213 participants in Tanzania to 9171 participants in The Gambia. The qualitative studies included 40 participants in Eritrea and 48 participants in Uganda.

The included studies were published between 2014 and 2024, with the greatest concentration in 2017 and 2024 (three studies each). No included studies were published in 2015 or 2022. All studies included adults aged 18 years or older. Multistage sampling was the most frequently reported sampling approach, followed by convenience sampling. Most studies were community‐based, with rural settings predominating. The characteristics of the included studies, including country, year of publication, study design, population, sample size, setting and key findings, are presented in Table 1.

### 3.2 Overview of Mapped Evidence

The synthesis of the 16 included studies identified 4 interconnected themes relating to hypertension‐related health‐seeking behaviour:

1. Patterns of healthcare‐seeking behaviour
2. Individual, socio‐economic and social determinants of healthcare‐seeking behaviour
3. Health‐system and sociocultural influences on healthcare seeking
4. Knowledge, awareness and health education as influences on care seeking

Medical pluralism, self‐medication, delayed care seeking and limited preventive screening emerged as important cross‐cutting issues across these themes.

### 3.3 Patterns of Hypertension‐Related Healthcare‐Seeking Behaviour

The included studies showed that adults with hypertension used a range of formal and informal healthcare options, reflecting a pluralistic pattern of healthcare‐seeking. Formal healthcare facilities, including hospitals, primary health centres and hypertension clinics, were commonly used, particularly by individuals who had been diagnosed with hypertension. These services were preferred because of their perceived quality, affordability, proximity and availability of trained healthcare professionals [24, 34]. However, access to formal healthcare did not always translate into consistent hypertension management. Azu et al. [9] found that contact with healthcare facilities did not necessarily result in blood pressure screening, whereas Faronbi et al. [16] reported that only a small proportion of participants sought hospital care after hypertension was detected.

Informal and alternative healthcare practices were also common. Participants sought care from patent medicine vendors, used over‐the‐counter medicines, consulted traditional healers and used herbal remedies [3, 16, 26]. These practices were influenced by cultural beliefs, perceived effectiveness, affordability, accessibility and experiences with formal healthcare. Traditional medicine was reported to be more common among rural populations [19], whereas self‐medication was also identified as an important component of hypertension‐related healthcare seeking [16, 29]. Some individuals used traditional or herbal remedies alongside prescribed medicines, whereas others discontinued biomedical treatment in favour of traditional remedies [17]. Prayer and other faith‐based approaches were also reported [25, 26].

Healthcare‐seeking patterns varied according to disease recognition and comorbidity. Tusubira et al. [44] found that individuals with both hypertension and diabetes were more likely to engage with formal healthcare and attend clinics regularly than those with hypertension alone. Bamgboye et al. [10] similarly reported that many diagnosed individuals initially sought formal care, although some subsequently used informal sources. Continued engagement with hypertension care was less consistent, particularly in relation to regular blood pressure monitoring and long‐term disease control.

Community and faith‐based approaches provided additional opportunities to strengthen healthcare engagement. Nonterah et al. [30] found that involving religious leaders improved hypertension awareness, screening uptake and linkage to care. Overall, the evidence indicates that hypertension‐related healthcare‐seeking is not confined to a single source of care. Rather, individuals navigate between formal, informal, traditional and community‐based options according to their perceived needs, resources, beliefs, accessibility and experiences of care.

### 3.4 Individual and Socio‐Economic Determinants of Healthcare‐Seeking Behaviour

Healthcare‐seeking behaviour was influenced by several individual and socio‐economic characteristics. Age, sex, education, income, employment, marital status and health insurance coverage were reported as important determinants of healthcare utilization across the included studies [9, 24, 34].

Higher income, stable employment, higher educational attainment and health insurance coverage were generally associated with greater use of formal healthcare services. In contrast, individuals with fewer socio‐economic resources were more likely to experience barriers to healthcare utilization and rely on informal sources of care. Faronbi et al. [16] similarly reported that older and wealthier participants were more likely to engage in regular blood pressure monitoring. These findings suggest that economic capacity and social position may influence not only whether individuals seek care but also the type and continuity of care they are able to access.

The asymptomatic nature of hypertension was another important individual level determinant. Because hypertension often produces no noticeable symptoms, individuals may perceive little need to seek care, particularly when they do not consider themselves ill. Tusubira et al. [44] reported that limited symptom recognition contributed to delayed care seeking, whereas Gebrezgi et al. [17] identified concerns about lifelong treatment, dietary restrictions and fear of complications as factors influencing engagement with care.

### 3.5 Social and Family Support

Social and family support emerged as an important facilitator of hypertension‐related healthcare seeking and treatment adherence. Gebrezgi et al. [17] and Karinja et al. [22] reported that encouragement from family members and social networks supported medication adherence, clinic attendance and engagement with recommended lifestyle practices. Family members could assist patients with reminders, transportation, medication use, clinic attendance and lifestyle changes. In some settings, however, social networks also contributed to the use of traditional remedies and informal sources of care.

The findings therefore suggest that social support can operate in both positive and negative directions, depending on the health beliefs and practices shared within families and communities.

### 3.6 Health‐System Determinants and Barriers to Care

Health‐system characteristics were repeatedly identified as important determinants of healthcare‐seeking behaviour. Availability and affordability of services, proximity to health facilities, availability of medicines, quality of provider patient relationships and respectful treatment by healthcare workers were reported as important facilitators [24]. Conversely, several studies identified medicine stock‐outs, long travel distances, financial barriers, inadequate health promotion, staff workload and limited screening services as barriers to sustained hypertension care [10, 17]. A particularly important finding was the presence of missed opportunities for hypertension detection within existing health services. Azu et al. [9] demonstrated that individuals could have contact with healthcare services without receiving blood pressure screening. This suggests that barriers to hypertension control are not solely related to whether people seek healthcare; the responsiveness and preventive capacity of health services also influence whether healthcare contact results in diagnosis and appropriate management.

### 3.7 Cultural Beliefs and Perceptions

Cultural beliefs and perceptions about hypertension strongly influence healthcare decisions. Studies reported misconceptions regarding the causes, severity, curability and treatment of hypertension. For example, beliefs that hypertension could be caused by spiritual forces, could be cured permanently or was primarily affected by older people were associated with delayed presentation and reliance on traditional or faith‐based treatment [27, 29].

These findings indicate that healthcare‐seeking behaviour cannot be understood solely in terms of availability or affordability of services. Individuals interpret hypertension through cultural and social frameworks that influence their decisions about when to seek care, where to seek care and whether to continue biomedical treatment.

### 3.8 Knowledge, Awareness and Health Education

Knowledge and awareness of hypertension varied considerably across the included studies. Several studies reported limited awareness of hypertension, its risk factors, complications and chronic nature. Lulebo et al. [27], for example, found that many participants were unable to identify important risk factors and complications. Bamgboye et al. [10] similarly reported substantial levels of previously undiagnosed hypertension, indicating gaps in population‐level awareness. Nonterah et al. [30] identified persistent misconceptions about the seriousness and curability of hypertension.

However, knowledge was not consistently associated with appropriate healthcare‐seeking behaviour. Faronbi et al. [16] found relatively good knowledge among university staff but identified substantial gaps between knowledge and preventive action. This suggests that knowledge alone may be insufficient to produce sustained healthcare‐seeking or preventive behaviour when financial, cultural, social or health‐system barriers remain present. In contrast, Gebrezgi et al. [17] found relatively good knowledge among patients attending hypertension follow‐up clinics, which was attributed partly to repeated health education and counselling. Nevertheless, misconceptions persisted, particularly regarding physical activity and lifestyle modification.

Overall, the findings indicate that health education may improve awareness and understanding, but its effect on healthcare‐seeking behaviour is likely to depend on the social and health‐system environment in which individuals make healthcare decisions.

### 3.9 Community and Faith‐Based Approaches to Improving Healthcare Engagement

Community‐based approaches emerged as an important strategy for strengthening hypertension awareness, screening and linkage to care. Nonterah et al. [30] demonstrated that engaging religious leaders could improve awareness and screening uptake and facilitate linkage to healthcare services. The findings across the review suggest that community structures, families, social networks and faith leaders can serve as important channels for hypertension education and early detection, particularly in settings where formal health services may be difficult to access or where cultural and religious beliefs strongly influence health decisions.

## 4 Discussion

This scoping review shows that health‐seeking behaviour related to hypertension prevention and management among adults in Africa is influenced by a range of individual, socio‐economic, social, cultural and health‐system factors. Across the 16 included studies, people used different sources of healthcare, including hospitals, primary healthcare facilities, pharmacies, medicine vendors, traditional healers, herbal remedies and faith‐based services. The findings suggest that seeking care for hypertension is not always a straightforward process. People may choose different sources of care depending on how they understand their condition, whether they feel they need care, what they can afford, how accessible services are, the advice they receive from family and community members and their previous experiences with healthcare. Healthcare‐seeking behaviour should therefore be understood within the wider social and healthcare environment in which people live.

A key finding from the review was the continued use of both formal and informal healthcare. Formal healthcare facilities were commonly used and were often preferred because they were seen as providing good quality care, being accessible and affordable and having trained healthcare professionals [24, 34]. However, using formal healthcare did not always mean that people received blood pressure screening or appropriate hypertension care. Some studies found that only a small proportion of participants sought formal care after identifying elevated blood pressure [9, 16]. At the same time, traditional medicine, herbal remedies, self‐medication and faith‐based care remained important sources of care [3, 19, 26, 29]. This suggests that people do not always see formal and informal healthcare as separate choices. Instead, they may use different sources of care at different points in their experience of hypertension. Evidence from outside Africa also supports this pattern, with [41] reporting the use of both conventional and complementary approaches among women managing hypertension. Overall, these findings suggest that healthcare seeking behaviour for hypertension management often involves moving between different sources of care rather than following one fixed pathway.

The way people understand hypertension may help explain this pattern. Hypertension is often described as a silent condition because many people do not experience symptoms, even when their blood pressure is high. This can make it difficult for people to recognize the need for regular monitoring and long‐term treatment. Several studies in the review found that people delayed seeking care until they experienced symptoms or developed complications [10, 44]. Similar findings have been reported outside Africa. Shrestha et al. [40], for example, found that some older adults in Nepal considered healthcare visits unnecessary when they had no symptoms and delayed routine monitoring until complications developed. Uddin et al. [45] also found that self‐treatment was common among hypertensive patients in Bangladesh, with some seeking formal care only when their condition became worse. These findings highlight the difficulty of managing a condition that may not make people feel ill but still requires regular monitoring and treatment.

Knowledge and awareness were also important in shaping healthcare‐seeking behaviour. Several studies found that limited knowledge about hypertension, its risk factors, complications and management was linked to delayed or inappropriate healthcare seeking [9, 10, 27, 30]. However, the review also showed that knowledge alone does not always lead to appropriate behaviour. Faronbi et al. [16], for example, found that although many Nigerian university staff had good knowledge of hypertension risk factors, blood pressure monitoring practices were still poor. Hasan et al. [18] similarly found that some hypertensive patients in Bangladesh sought care from informal providers even though they were aware of their condition. In contrast, Gebrezgi et al. [17] found that repeated counselling and follow‐up improved knowledge among hypertensive patients, whereas Pirouz et al. [37] and Siraj et al. [42] identified awareness and education as important factors associated with healthcare‐seeking behaviour. These findings suggest that knowledge is important, but it is only one part of the decision‐making process. What people know about hypertension may not translate into action when other challenges, such as cost, access, social influence, cultural beliefs or problems within the health system, make it difficult to act on that knowledge. This points to an important gap between knowledge and behaviour.

The socio‐economic circumstances in which people live further influence their healthcare decisions. According to the reviewed studies, higher education, stable employment, health insurance and higher household income were generally associated with greater use of formal healthcare services [9, 24, 26, 34]. In contrast, financial difficulties often made it harder for people to seek care or continue treatment. The cost of medicines, transportation and healthcare services could lead to delays in seeking care or greater reliance on less costly sources of treatment. Abdulai et al. [2], for example, identified transportation and medication costs as important barriers among Ghanaian patients living with HIV and hypertension. Hasan et al. [18] found that poorer hypertensive patients in Bangladesh were more likely to use informal providers, whereas Chakraborty et al. [11] reported a greater financial burden of hypertension management among poorer households in India. These findings show that healthcare‐seeking behaviour is not simply a matter of personal choice. People's decisions are also shaped by what they can afford and what services are realistically available to them.

Family and social networks were another important part of the healthcare‐seeking process. Support from family members and others close to the individual could encourage medication adherence, clinic attendance and healthier lifestyle choices [17, 22]. Family members could also provide emotional and financial support, which may be particularly important for a condition requiring lifelong treatment [2]. However, social influence did not always encourage formal healthcare use. Family members, friends and community networks could also recommend traditional remedies, herbal medicines or faith‐based care. This suggests that social support can influence not only whether people seek care but also where they seek it. Understanding these social influences is therefore important when developing hypertension interventions.

The healthcare system itself also shaped people's decisions. Even when individuals recognized the need for care, problems within the health system could affect whether they were able to obtain it. The reviewed studies identified medicine shortages, staff shortages, distance to healthcare facilities, cost, waiting times and concerns about the availability and quality of services as important barriers [10, 17, 24]. Similar challenges have been reported in Kenya, Bangladesh, Nepal and Ethiopia [36, 40, 42, 45]. These findings suggest that improving healthcare‐seeking behaviour cannot rely on educating individuals alone. People also need healthcare services that are accessible, affordable, reliable and able to provide medicines and follow‐up care when they seek treatment.

Cultural and spiritual beliefs also influenced how people understood hypertension and where they sought care. Several studies reported misconceptions about the causes, severity, curability and management of hypertension [27, 29, 30]. Some participants believed that hypertension could be permanently cured or linked to supernatural causes such as witchcraft, curses or spiritual punishment. Atibila et al. [8], for example, reported such beliefs among some participants in Ghana. These beliefs may influence whether people seek formal healthcare, use traditional remedies or turn to faith‐based care. They may also help explain why some people move between different sources of healthcare. This does not mean that cultural beliefs should simply be treated as barriers. Rather, it highlights the need for healthcare interventions that understand people's beliefs and experiences while providing clear and culturally appropriate information about hypertension.

The review also identified community‐based approaches as an important opportunity for improving hypertension prevention and management. Religious leaders, community leaders and other trusted community members can play an important role in reaching people who may not regularly use formal healthcare services. Nonterah et al. [30], for example, found that involving religious leaders improved hypertension awareness, screening, linkage to care and treatment adherence. Community‐based approaches may therefore provide an effective way of increasing awareness, encouraging blood pressure screening, supporting referral and promoting healthy behaviours. However, these approaches should complement formal healthcare rather than replace it.

Overall, the findings tell a consistent story about how people make decisions about hypertension care. Healthcare seeking is influenced not only by what people know about hypertension but also by whether they recognize the need for care, whether they can afford and reach services, what their families and communities recommend, how they understand the condition, and what they experience when they use healthcare services. Formal healthcare remains an important source of care, but many people also use informal sources, sometimes before, alongside or instead of formal healthcare. Healthcare seeking behaviour for hypertension management is therefore better understood as a process in which people may move between different sources of care according to their circumstances and needs.

These findings have important implications for hypertension prevention and management in Africa. Interventions that focus only on improving knowledge are unlikely to be enough. Greater attention is needed to the social and economic conditions that shape healthcare decisions, the accessibility and reliability of health services, the role of families and communities and the cultural beliefs surrounding hypertension. There is also a need to better understand how people navigate and combine formal and informal healthcare in managing the condition. Understanding this process may provide important insights into delayed care, treatment adherence and long‐term hypertension management.

### 4.1 Research Gaps

Several important gaps were identified across mapped literature. The evidence was geographically uneven, with studies concentrated in a limited number of African countries and limited representation from several regions of the continent. Most studies were institution‐based and focused on individuals who had already been diagnosed with hypertension or were interacting with healthcare services. As a result, there was limited evidence on health‐seeking behaviour related to hypertension prevention, routine blood pressure screening and early detection among the general adult population, particularly those who had not yet been diagnosed.

The evidence was also mainly based on cross‐sectional quantitative studies, with relatively few qualitative and mixed methods exploring the social, cultural and personal factors that influence health‐seeking decisions. This limited understanding of how health‐seeking behaviour changes over time and how individual experiences influence treatment initiation, medication adherence and long‐term hypertension management. Although family support, social networks, faith‐based engagement and traditional healing were identified as important influences, there was limited understanding of how these factors shape healthcare decisions and continued engagement with treatment.

Although the use of different sources of healthcare was reported, the included studies provided limited evidence on why adults with hypertension use different forms of care, how they navigate between conventional and traditional treatments, and how these treatments are combined in managing hypertension. Evidence comparing these practices across rural and urban populations was also limited. In addition, although medication adherence was reported in some studies, there was limited evidence on the socio‐demographic and health belief model factors associated with adherence. These gaps highlight the need to examine both medication adherence and its determinants, as well as the use and navigation of conventional and traditional treatments across rural and urban communities among adults with hypertension in Africa.

Another important gap concerns the relationship between knowledge and actual health‐seeking behaviour. Several studies showed that knowledge or awareness of hypertension did not always lead to appropriate preventive practices, timely healthcare seeking or treatment adherence. However, there was limited evidence on why this knowledge–behaviour gap exists or how it can be addressed effectively.

Overall, the evidence highlights the need for more geographically diverse, community‐based, qualitative and mixed‐methods research on health‐seeking behaviour across the hypertension prevention and management continuum. Future research should also examine how individual, socio‐economic, social, cultural and health‐system factors influence healthcare decisions and how people navigate and combine formal and informal healthcare services.

### 4.2 Implication for Practice

The findings have important implications for clinical practice and public health.

**Clinical practice**: Healthcare providers should routinely ask patients about their use of informal and non‐conventional healthcare, including traditional medicines, herbal remedies, self‐medication and faith‐based care. This can help identify possible risks and ensure that patients receive appropriate advice. Patient counselling should also emphasize that hypertension is often asymptomatic, the importance of regular blood pressure monitoring, the need for lifelong treatment adherence and the risks of uncontrolled hypertension and its complications. Where appropriate, family members should be involved in hypertension management to provide support for treatment and healthy lifestyle practices. Blood pressure measurement should also be integrated into routine healthcare encounters, including opportunistic screening, to improve early detection.

**Public health**: Community‐based hypertension screening programmes should be strengthened to improve early detection and referral. Access to affordable antihypertensive medicines should be improved, whereas financial and transport barriers that prevent people from accessing care should be reduced. Primary healthcare services should be strengthened to support long‐term hypertension treatment, regular follow‐up and medication adherence.

Community engagement should also be strengthened. Religious leaders, community leaders and community health workers can play an important role in increasing awareness, encouraging blood pressure screening, supporting referral and promoting healthy behaviours. Interventions should be culturally sensitive and should address common misconceptions about hypertension while promoting evidence‐based prevention and management. Overall, hypertension interventions should combine clinical care with community‐based approaches to improve early detection, treatment and long‐term disease management.

### 4.3 Strength and Limitation

First, it only included published studies from African countries. This may limit the generalizability of the findings to other regions with different healthcare systems, cultures and economic conditions.

Second, a limitation of the search strategy is that it did not comprehensively include all individual African country names in the search string. Although broad regional terms such as ‘Africa’ and ‘Sub‐Saharan Africa’ were used to capture studies across the continent, the omission of some country‐specific keywords may have resulted in the exclusion of relevant studies indexed under specific national contexts. Given that database indexing practices vary and many studies are catalogued primarily by country name, this approach may have reduced the sensitivity of the search and introduced a risk of missing eligible literature from certain settings.

Third, the review only included studies written in English. This may have led to the exclusion of relevant studies published in other languages, meaning that important evidence from non‐English‐speaking settings may have been missed.

Fourth, there may be publication bias, as studies with clear or positive findings are more likely to be published than those with unclear or negative results. This could have influenced the overall findings of the review.

Finally, the included studies varied in design, sample size and methodology, which made direct comparison across challenging studies. However, the review still provides a useful synthesis of common patterns in health‐seeking behaviour for hypertension in Africa.

### 4.4 Future Research

Future research should address the gaps identified in this review. First, more community‐based studies are needed in African countries and regions that are not well represented in the current evidence. These studies should include adults who have not been diagnosed with hypertension. This would improve understanding of prevention, blood pressure screening, early detection and healthcare‐seeking behaviour before diagnosis.

Second, more qualitative and mixed‐methods studies are needed to understand why people make different healthcare decisions. Future research should explore how people decide where to seek care, why they move between conventional and traditional medicine and why some people combine both approaches when managing hypertension.

Third, more research is needed to understand differences between rural and urban populations. Comparative studies could examine differences in healthcare access, healthcare preferences, socio‐economic conditions, social support, cultural beliefs and health‐system factors.

Fourth, longitudinal studies are needed to understand how health‐seeking behaviour changes over time. Research should examine the different stages of hypertension care, from prevention and screening to diagnosis, treatment, medication adherence, follow‐up and long‐term management.

Finally, future research should examine the gap between knowledge and behaviour. Studies should explore why knowledge and awareness of hypertension do not always lead to healthy practices, timely healthcare seeking or treatment adherence. Research should also identify effective ways to help people turn their knowledge into sustained healthy behaviours. Future studies should consider how individual, socio‐economic, social, cultural and health‐system factors influence these behaviours.

## 5 Conclusion

The evidence demonstrates that healthcare‐seeking behaviour among adults with hypertension in Africa is shaped by individual, socio‐economic, cultural and health‐system influences. Formal healthcare facilities remain important sources of care, yet many individuals continue to rely on traditional healers, herbal remedies, self‐medication, medicine vendors and faith‐based sources of support. Knowledge and awareness are important determinants of healthcare‐seeking behaviour, but they are insufficient on their own to ensure early diagnosis, treatment initiation, adherence or long‐term disease control. Financial constraints, accessibility barriers, cultural beliefs, social support systems and health‐system weaknesses all influence hypertension‐related healthcare decisions. Improving hypertension outcomes therefore requires comprehensive interventions that address these determinants while strengthening community engagement, healthcare accessibility, affordability and continuity of care. Future research should prioritize formal and informal healthcare studies, urban‐rural comparisons, preventive healthcare‐seeking behaviours and the influence of social and cultural factors on hypertension management across diverse African settings.

## Author Contributions

Mustapha Sanyang was responsible for the design of the study, literature search, data extraction, analysis and writing of the manuscript. Hafiz T. A. Khan and Salim Vohra provided guidance throughout the study process and contributed to ensuring the quality and rigour of the manuscript. All authors read and approved the final version of the manuscript.

## Funding

This study was supported by the University of West London through the Vice Chancellor's Scholarship. No additional funding was received from public, commercial or not‐for‐profit agencies.

## Ethics Statement

This scoping review is part of my PhD studies at the University of West London. Ethical clearance for this research was obtained from the University of West London Research Ethics Committee and the Joint MRC Gambia Government Ethics Committee. The review was conducted using secondary data from published peer‐reviewed sources. All materials were properly cited to maintain academic integrity, and the review process adhered to ethical standards for research integrity and transparency as outlined by both institutions.

## Consent

The authors have nothing to report.

## Conflicts of Interest

The authors declare no conflicts of interest.

## Data Availability

The data that support the findings of this study are available in the Supporting Information of this article.
